# Bilayer Membrane Modulation of Membrane Type 1 Matrix Metalloproteinase (MT1-MMP) Structure and Proteolytic Activity

**DOI:** 10.1038/srep29511

**Published:** 2016-07-13

**Authors:** Linda Cerofolini, Sabrina Amar, Janelle L. Lauer, Tommaso Martelli, Marco Fragai, Claudio Luchinat, Gregg B. Fields

**Affiliations:** 1 Giotto Biotech S.R.L., Via Madonna del Piano 6, 50019 Sesto Fiorentino (FI), Italy; 2 Department of Chemistry & Biochemistry, Florida Atlantic University, 5353 Parkside Drive, Jupiter, FL 33458, USA; 3 Max Planck Institute of Molecular Cell Biology and Genetics, Pfotenhauerstrasse 108, 01307, Dresden, Germany; 4 CERM, University of Florence, Via Luigi Sacconi 6, 50019, Sesto Fiorentino (FI), Italy; 5 Department of Chemistry “U. Schiff”, University of Florence, via della Lastruccia 3, 50019, Sesto Fiorentino (FI), Italy; 6 Department of Chemistry, The Scripps Research Institute/Scripps Florida, 130 Scripps Way, Jupiter, FL 33458, USA; 7 Departments of Chemistry and Biology, Torrey Pines Institute for Molecular Studies, 33458, Port St. Lucie, FL 34987, USA

## Abstract

Cell surface proteolysis is an integral yet poorly understood physiological process. The present study has examined how the pericellular collagenase membrane-type 1 matrix metalloproteinase (MT1-MMP) and membrane-mimicking environments interplay in substrate binding and processing. NMR derived structural models indicate that MT1-MMP transiently associates with bicelles and cells through distinct residues in blades III and IV of its hemopexin-like domain, while binding of collagen-like triple-helices occurs within blades I and II of this domain. Examination of simultaneous membrane interaction and triple-helix binding revealed a possible regulation of proteolysis due to steric effects of the membrane. At bicelle concentrations of 1%, enzymatic activity towards triple-helices was increased 1.5-fold. A single mutation in the putative membrane interaction region of MT1-MMP (Ser466Pro) resulted in lower enzyme activation by bicelles. An initial structural framework has thus been developed to define the role(s) of cell membranes in modulating proteolysis.

The regulation of biological activity at the cell surface is achieved through a variety of processes including recycling, shedding, and protein-protein and protein-membrane interactions. While protein activities are often studied in isolation, methods by which activities could be evaluated in membrane-like environments provide greater insight into *in vivo* behaviors. Membrane type 1 matrix metalloproteinase (MT1-MMP) is a type I transmembrane cell-surface protease that has been implicated in numerous pathologies^1^,^2^,^3^. MT1-MMP possesses a multidomain structure, encompassing a signal peptide, propeptide, catalytic (CAT) domain, hinge region (linker 1), hemopexin-like (HPX) domain, stalk region (linker 2), transmembrane domain (TM), and cytoplasmic tail (CT) (Fig. 1). Active MT1-MMP (Fig. 1) has been ascribed a variety of cell surface activities, with one of the most prominent being that of a pericellular collagenase. MT1-MMP collagenolytic activity appears critical for transmigration of tumor cells, endothelial cells, and fibroblasts through collagen matrices^4^,^5^,^6^,^7^,^8^,^9^, while post-myocardial infarction survival has been correlated to the collagenolytic potential of cardiac fibroblasts, where MT1-MMP is the dominant collagenase within myocardial tissues^10^.

The plasma membrane environment provides a unique milieu for the regulation of MT1-MMP collagenolytic activity^2^. However, the manner in which bilayers interact with MT1-MMP has not been examined. A recent study found specific interactions of MMP-12 with bicelles and cells^11^. In order to investigate the influence of bilayers on MT1-MMP behavior, structure and activity analyses of MT1-MMP in membrane-like environments are desired. Different membrane-mimicking environments, such as micelles, liposomes, and bicelles, have been used in structural studies^12^. In particular, bicelles, constituted by long-chain phospholipids and short-chain phospholipids or detergents, as well as liposomes, have been observed to mimic the natural membrane better than standard micelles^13^.

At present, structural data on MT1-MMP is limited. The X-ray crystallographic structure of the MT1-MMP CAT domain, in complex with tissue inhibitor of metalloproteinases (TIMPs), has been reported (pdb 3MA2, 1BUV, 1BQQ)^14^,^15^. An X-ray crystallographic structure of the MT1-MMP HPX domain homodimer has been solved (pdb 3C7X)^16^, as has an NMR spectroscopic structure for the binding of collagen-model triple-helices to the MT1-MMP HPX domain (pdb 2MQS)^17^. There are no structures of the intact soluble MT1-MMP ectodomain (CAT domain-linker1-HPX domain, see Fig. 1) or structures of MT1-MMP interacting with a bilayer membrane.

The kinetics for soluble MT1-MMP ectodomain catalysis of triple-helices has been reported^17^,^18^,^19^,^20^, but data for membrane-bound MT1-MMP processing of triple-helices has not. As collagenolysis is one of the integral MT1-MMP activities, we presently characterized membrane-bound MT1-MMP ectodomain in the presence and absence of a triple-helical substrate by NMR spectroscopy and FRET kinetic assays. We have also examined the MT1-MMP S466P mutant, previously shown to effect mouse development resulting in the “cartoon mouse” 21, to evaluate the effect of the mutation on bilayer interaction and enzyme kinetics.

## Results

**Figure 2:**
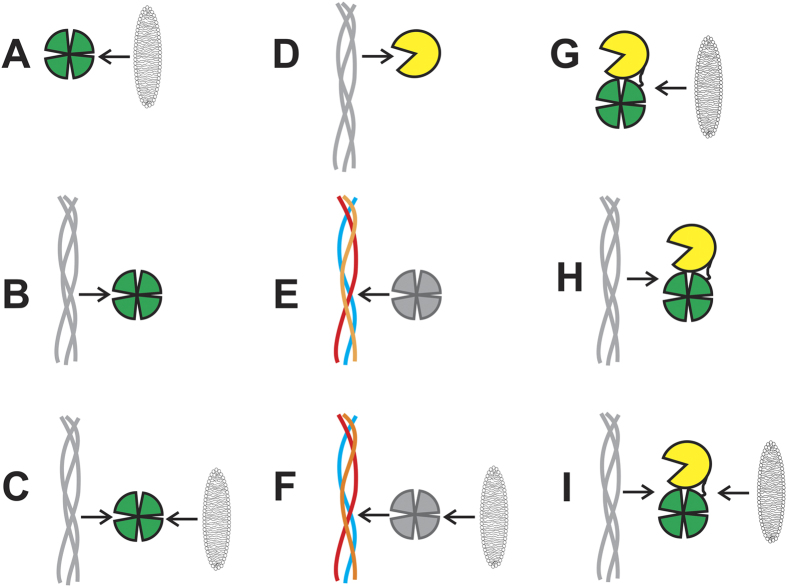
Interaction complexes examined by NMR spectroscopy. (**A**) Labeled MT1-MMP HPX domain with bilayer mimics; (**B**) labeled MT1-MMP HPX domain with THP; (**C**) labeled MT1-MMP HPX domain with bilayer mimics and THP; (**D**) labeled MT1-MMP CAT domain with THP; (**E**) MT1-MMP HPX domain with labeled THP; (**F**) MT1-MMP HPX domain with bilayer mimics and labeled THP; (**G**) labeled sMT1-MMP with bilayer mimics; (**H**) labeled sMT1-MMP with THP; and (**I**) labeled sMT1-MMP with bilayer mimics and THP.

The isolated CAT and HPX domains of MT1-MMP, as well as the soluble ectodomain of MT1-MMP (sMT1-MMP hereafter), were expressed and the resonances assigned by NMR (Figures S1–S3). Relaxation measurements were performed on the individual domains and on the sMT1-MMP construct at 298 K (Figure S4). The average of the transverse relaxation rate (R_2_) values for sMT1-MMP appeared to be sizably lower with respect to the theoretical values for the rigid protein structure expected from X-ray data on MMP-12^22^ and modelled with HydroNMR^23^. Lower R_2_ values indicated that sMT1-MMP behaved as a lower molecular weight protein. As previously observed in MMP-1, this occurs when the CAT and HPX domains are not rigidly held, but can reorient with respect to one another^24^,^25^. The interaction of the soluble ectodomain of MT1-MMP and of its isolated hemopexin-like domain with membrane bilayers and with a collagen-like triple helical peptide (THP) was investigated in detail by 2D ^1^H-^15^N HSQC NMR spectra (Fig. 2). Also the interaction of the isolated CAT domain with THP was analyzed (Fig. 2D). The collagen-like triple-helical peptide was the same previously used to investigate the mechanism of collagenolysis in MMP-1^24^.

### Interaction of the isolated ^15^N MT1-MMP HPX domain with bilayers

**Figure 3:**
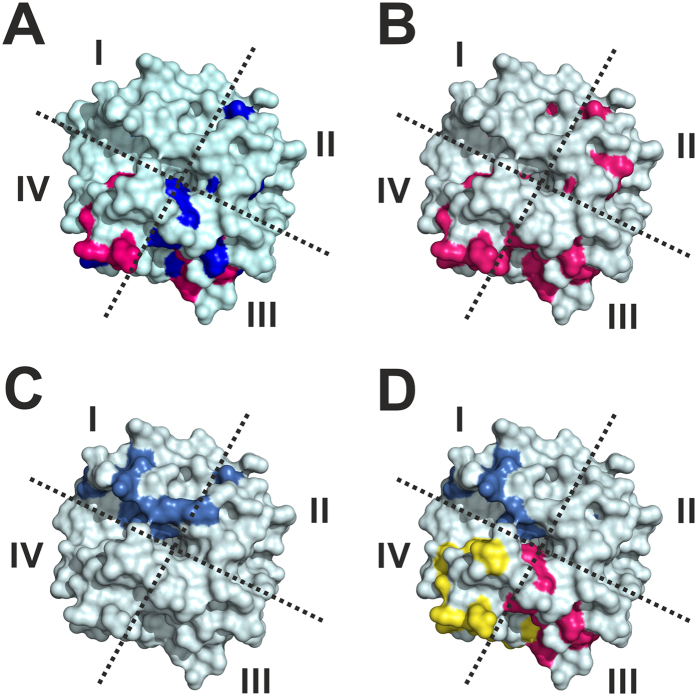
Surface representation of the MT1-MMP HPX domain with the residues exhibiting the largest decrease in NMR signal intensity in the presence of: (**A**) 1% w/v bicelles (the residues interacting selectively with bicelles are colored in magenta, while those affected also by the addition of CHAPS are colored in blue); (**B**) 10 μL of pellet containing 2.0 × 10^6^ HEK293T cells (in magenta); (**C**) THP (molar ratio HPX:THP = 1:0.5) (in blue): (**D**) 1% w/v bicelles and THP (molar ratio THP:HPX = 1:1) (in magenta and blue, respectively). The residues affected by the binding to bicelles, whose signal intensity increases with the addition of THP, have been highlighted in yellow in panel D.

The isolated HPX domain was initially studied, as this domain alone binds type I collagen^26^. The intensity changes per residue of the HPX domain were evaluated in the presence of 1% w/v bicelles (Figure S5A). The residues exhibiting the largest decrease in signal intensity are localized in blades III and IV of the β-propeller (Fig. 3A), with some residues also exhibiting a small chemical shift variation (Figure S6A). Small chemical shift perturbations were also observed for a few residues in blades I and II. At 0.5% w/v bicelles, very few changes in signal intensity were observed, while at 2 and 4% w/v bicelles, the signal intensity changes were very similar to 1% w/v ratio. Collectively, these data show that bicelles interact selectively with specific residues of blades III and IV (Fig. 2A).

Titration of the HPX domain with DPPC liposomes (0.3, 1, 1.7, 2.3, and 3% w/v) resulted in a generalized decrease in signal intensity, without any sizable selectivity (Figure S7A). This behavior is in contrast to what was observed in the presence of bicelles which interact with a well-defined region of the β-propeller. In addition, a few residues (Figure S7B) experienced very small chemical shift variations upon the addition of liposomes.

The intensity changes per residue of the HPX domain were subsequently evaluated in the presence of human embryonic kidney (HEK293T) cells to investigate the interaction of this domain with the cellular membrane. 2D ^1^H-^15^N SOFAST-HMQC experiments were performed on the HPX domain before and after the addition of 10 μL of pellet containing 2.0 × 10^6^cells. The pattern of residues exhibiting the largest decrease in signal intensity is similar to that observed following addition of bicelles to the protein (Figure S8) and involves blades III and IV of the β-propeller (Fig. 3B). MT1-MMP has been proposed to have cell surface binding partners that interact with HPX domain blades III and IV, and the cell interactions identified herein may impact these binding events (see Discussion).

### Interaction of the isolated ^15^N MT1-MMP HPX domain with 3-[(3-cholamidopropyl)dimethylammonio]-1-propanesulfonate (CHAPS)

High critical micelle concentration (CMC) values of the detergents used to prepare bicelles have been reported to be a limiting factor for the use of these bilayer assemblies as model membranes^27^,^28^,^29^. When the CMC value is high, the detergent used to prepare the bicelles, can be present in solution at a relatively high concentration as free monomer, and it can interact with the protein. This makes it difficult to analyze the effects of bicelles on the protein surface. As a result, the use of bicelles containing detergent with a low CMC value is emerging as a new strategy^30^. Alternatively, the protein can be titrated with the detergent alone to discriminate between the effects of bicelles and those of the free detergent. Therefore, the HPX domain was titrated with increasing concentrations of CHAPS, and the effects on the protein residues were monitored by NMR.

The intensity change and the chemical shift variation per residue of the HPX domain in the presence of 3.2 mM CHAPS were evaluated (Figure S9A,B). This concentration corresponds to the total amount of CHAPS (as free monomer and in bicelles) present in solution when 1% w/v of bicelles are added to the protein sample. The residues experiencing an effect in the presence of CHAPS alone are marked in the histogram reproducing the decrease in signal intensity after the addition of bicelles (Figure S10). Interestingly, most of the residues in blades I and II, which experience chemical shift variation in the presence of bicelles (R330, V344, I357, Q359, F360, and A393), are observed to experience a similar effect in the presence of CHAPS alone. This finding suggests that the effects observed in blades I and II are not related to the interaction with bicelles. Protein unfolding was observed in the presence of CHAPS at concentrations higher than 3.2 mM.

### Interaction of the isolated ^15^N MT1-MMP HPX domain with THP

The interaction of the THP with the HPX domain was evaluated at different HPX:THP molar ratios (Figure S5B). In this case, the residues exhibiting the largest decrease in signal intensity are localized in blades I and II of the β-propeller (Fig. 3C). It is immediately apparent that the region of the isolated HPX domain interacting with the THP is different from the protein surface interacting with bicelles (Fig. 3A), and involves residues localized in blades I and II (Fig. 2B).

### Interaction of the isolated ^15^N MT1-MMP HPX domain with THP in the presence of bicelles

The HPX domain intensity changes per residue for different HPX:THP molar ratios in the presence of bicelles were analyzed in detail (Figure S5C). The residues exhibiting the strongest decrease in signal intensity were largely the same as those observed with the HPX alone (Figs 2C and 3A,D).

In contrast to what was previously observed in the absence of bicelles, several residues exhibited a chemical shift variation upon the addition of THP (Figure S6B). It is interesting to note that most of the residues experiencing a chemical shift variation upon the addition of bicelles were further shifted by the THP.

The residues that experienced a decrease in signal intensity by addition of THP, in the absence of bicelles, show the same behavior upon further addition of bicelles (Figs 3C,D and S5). These data demonstrate that the THP binds the same region of the HPX domain in the presence and absence of bicelles. At the same time, some residues involved in the interaction with bicelles experience an increase of the signal intensity when the THP is added to the protein in the presence of bicelles (Figs 3D and S5). Therefore, interaction of the HPX domain with THP seems to alter the interaction of the four-bladed β-propeller with bicelles.

### Interaction of the isolated ^15^N MT1-MMP CAT domain with THP

The interaction of the THP with the isolated CAT domain was evaluated at different CAT:THP molar ratios. However, the effect of the addition of the THP to the MT1-MMP CAT domain was negligible and no chemical shift variations or intensity decreases were observed. A weak affinity of the isolated CAT domain for THP was also observed in MMP-1^24^ (Fig. 2D).

### Interaction of the ^13^C,^15^N THP with MT1-MMP HPX

**Figure 4:**
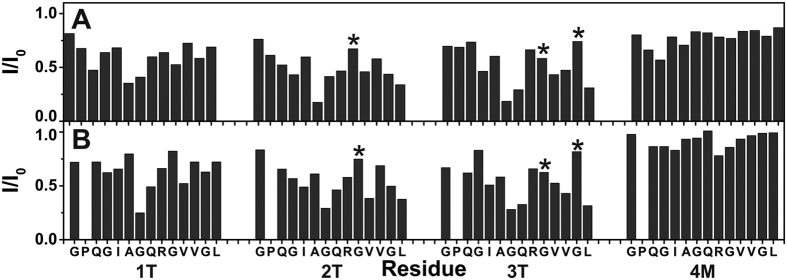
Intensity changes per residue of ^13^C,^15^N isotopically enriched THP in the presence of the MT1-MMP HPX domain (molar ratio THP:HPX = 1:0.2) (panel A), and MT1-MMP HPX domain (molar ratio THP:HPX = 1:0.2) with 1% w/v bicelles (panel B). The individual chains of the THP are, from left to right, 1T, 2T, and 3T, while the far right is the monomeric strand 4M. Identification of the individual THP strands was described previously^24^. The stars indicated residues in overlap for which the measure of intensity is less precise or reliable.

To identify the residues of THP involved in the binding of MT1-MMP, the isolated HPX domain was added to ^13^C,^15^N isotopically enriched THP. Analysis of the intensity changes per residue of the THP (Fig. 4A) revealed that chains 2T and 3T of the triple-helix interact more with HPX than chain 1T. For example, 2T and 3T showed significant decreases in intensity for L26, while 1T had no intensity decrease for this residue. There was also a larger decrease for the intensity of G19 in 2T and 3T compared with 1T. The interactions with the MT1-MMP HPX domain are thus different from those observed in MMP-1. MMP-1 interacts with residues closer to the *C*-terminus of the THP (V23, V24, G25, and L26), while exhibiting no interaction with residues near the site of hydrolysis (for example, G19 and Q20)^24^ (Fig. 2E).

### Interaction of the ^13^C,^15^N THP with MT1-MMP HPX in the presence of bicelles

The intensity changes per residue of isotopically enriched THP in the presence of 1% w/v bicelles were analyzed for different THP:HPX molar ratios. The residues exhibiting the largest decrease in signal intensity were G19 in 1T, G19, Q20, V23, and L26 in 2T, and G19, Q20, V24, and L26 in 3T (Fig. 4). Clearly, the THP interacts with the HPX domain in the same manner both in the presence and absence of bicelles (Fig. 2F).

### Interaction of ^15^N sMT1-MMP with bicelles

**Figure 5:**
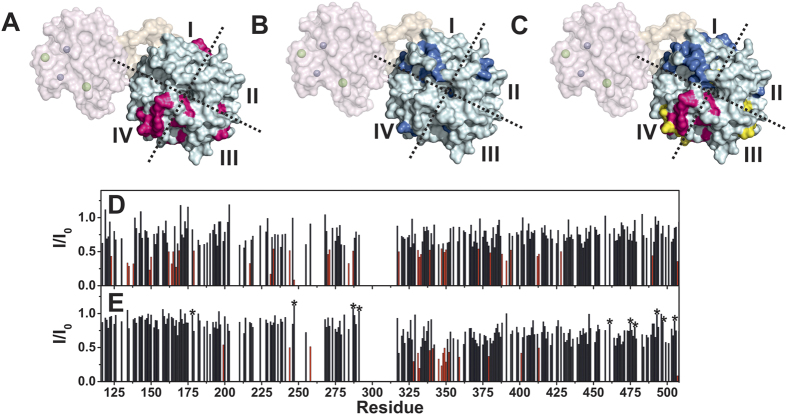
Top: Surface representation of a homology model of sMT1-MMP with the residues of the HPX domain exhibiting the largest decrease in NMR signal intensity in the presence of (**A**) 2% w/v bicelles (in red), (**B**) THP (molar ratio sMT1-MMP:THP = 1:0.4) (in blue), or (**C**) 2% w/v bicelles and THP (molar ratio sMT1-MMP:THP = 1:2) (in red and blue, respectively). The residues affected by the binding to bicelles, whose signal intensity increases with the addition of THP, have been highlighted in yellow in panel C. Bottom: Intensity changes per residue of sMT1-MMP in the presence of THP (molar ratio sMT1-MMP:THP = 1:0.4) (**D**) and 2% w/v bicelles and THP (molar ratio sMT1-MMP:THP: = 1:2) (**E**). The residues exhibiting the largest decrease in signal intensity in the presence of THP (E123, K134, V135, Y138, R149, V150, V162, Y164, A165, I167, E169, I179, S217, N231, I233, A244, L247, V270, L271, G284, S287, I318, G331, E332, M333, R339, N347, V349, M350, Y372, V380, W388, D391, S394, T412, D413, Y428, K490, G507), and bicelles and THP (L199, A244, A258, M328, G331, E332, R339, F341, R345, N347, Q348, V349, M350, G352, Q359, F379, K401, D413, G469 and G507) have been highlighted in red. After the addition of bicelles some residues in the CAT domain and in blades III and IV of the HPX domain exhibited a decrease in signal intensity (I177, G211, A244, L247, A255, A258, G285, and K292 in the CAT domain and A327, Q359, K401, L406, W421, M422, Y435, L442, N452, K454, E461, R464, G469, D471, V473, T475, Y478, N487, Q489, K490, K492, E494, K499, and M506 in the HPX domain). The intensity of the signals belonging to sMT1-MMP before the addition of bicelles and THP was taken as reference (intensity = 1). The residues exhibiting an increase in signal intensity after the addition of THP in the presence of bicelles have been marked with a star.

The effect of increasing amounts of bicelles (0.5, 1, and 2% w/v) on sMT1-MMP was analyzed. Several residues within blades III and IV of the HPX domain experienced a sizable decrease of the signal intensity in the presence of bicelles (2% w/v) (Figs 2G and 5A).

### Interaction of ^15^N sMT1-MMP with THP

The effect of increasing amounts of THP on sMT1-MMP was investigated at different sMT1-MMP:THP molar ratios (Fig. 5D). The addition of the THP to the ^15^N isotopically enriched sMT1-MMP sample induces a generalized decrease of the signal intensity of the residues of the ectodomain. However, the residues of the HPX belonging to blades I and II, and observed to bind THP when the isolated HPX domain is present in solution, experienced the largest effects (Figs 2H and 5B,D).

### Interaction of ^15^N sMT1-MMP with THP in the presence of bicelles

To evaluate how the membrane-mimicking bilayer and the collagen model interplay with the ectodomain, THP was added to sMT1-MMP in the presence of 2% w/v bicelles. The effect of increasing amounts of THP was evaluated at different sMT1-MMP:THP molar ratios. Collectively, the experimental data indicate that in the presence of bicelles the pattern of residues involved in the THP interaction is largely the same (Fig. 5C,E) as in the absence of bicelles (Fig. 5B,D). Further, the HPX domain residues involved in the interaction with THP are approximately the same in the isolated HPX domain and in sMT1-MMP (Fig. 2I).

An analysis of chemical shift variations upon the addition of THP to the sMT1-MMP indicated that most of the residues experiencing a chemical shift variation upon sMT1-MMP interaction with bicelles were further shifted by the addition of the THP (Figure S11). This suggests that the interaction of the HPX domain with the THP slightly alters the interaction of the HPX domain with bicelles, as observed with the isolated HPX domain (Fig. 3D).

The NMR data were used to calculate a structural model reproducing the transient interaction of the HPX domain with the membrane-mimicking bilayer (Figure S12A,C). The residues experiencing the largest variation in signal intensity were used as “active residues” in docking calculations with a membrane model. The same NMR data were then used to calculate a structural model between the HPX•THP complex recently reported (pdb: 2MQS) and the membrane (Figure S12B,D). The role of the residues experiencing a decrease in signal intensity in the presence of CHAPS alone was analyzed by performing different sets of docking calculation in which they were included (Figure S12A,B) or excluded (Figure S12C,D) as “active residues”. It is interesting to note that in the docking models generated starting from the two distinct sets of data, the HPX domain binds the membrane in a similar fashion.

### sMT1-MMP hydrolysis of THP in the absence or presence of bilayer mimics

The hydrolysis of a FRET analog of the THP (fTHP-9 hereafter) by sMT1-MMP was examined for increasing concentrations of bicelles or liposomes (Table 1 and Figure S13). Enzyme activity was substantially increased in 1% bicelles, and only slightly increased in 5% bicelles. The increase was entirely due to an increased k_cat_ value. 20% bicelles decreased enzyme activity. In contrast, 1% liposomes increased enzyme activity to a much lower degree than 1% bicelles. 5% liposomes had little effect on activity, while 20% liposomes decreased activity.

We next sought to examine the effect of a mutation in the putative membrane interacting region of the HPX domain. In the course of confirming an immunological phenodeviant that resulted in the “cartoon mouse”, a Ser466 to Pro MT1-MMP mutation was recovered serendipitously^21^. This mutation is within one of the HPX domain regions that interacts with bicelles (see Figures S5A and S12). To evaluate the effects of this mutation on MT1-MMP functions, sMT1-MMP S466P was expressed and purified. Circular dichroism (CD) analysis of sMT1-MMP and sMT1-MMP S466P indicated small differences in overall structure between the two proteins, but nothing to suggest substantial differences in protein folding (Figure S14). The activity of sMT1-MMP S466P was higher than sMT1-MMP, entirely due to k_cat_ (Table 1). However, the activity of sMT1-MMP S466P in the presence of 1% bicelles was lower than sMT1-MMP (Table 1 and Figure S13). In the bicelle, k_cat_ was much lower for sMT1-MMP S466P than for sMT1-MMP (Table 1 and Figure S13). The opposite trend was observed in liposomes, where 1% liposomes produced higher activity in sMT1-MMP S466P compared with sMT1-MMP (Table 1 and Figure S13). Thus, the mutation of Ser466 to Pro effected MT1-MMP interaction with bilayer membranes and subsequent triple-helical peptidase activity. The mode of inhibition by 20% bicelles or liposomes, for both enzymes, was non-competitive or mixed.

## Discussion

Prior studies on collagenolytic mechanisms found that the MMP-1 HPX domain initiates collagen binding, followed by the CAT domain being brought into the proximity of the triple-helix^22^,^24^,^25^,^31^,^32^,^33^,^34^,^35^. MT1-MMP appears analogous in this regard, as the mode of triple-helix binding to the HPX domain and sMT1-MMP was similar, while binding to the isolated CAT domain was weak at best. However, once in the proximity of the triple-helix, binding sites within the CAT domain (outside of the active site) were observed. In particular, residues 218–233 of the V-B loop are impacted by THP binding. This loop has been previously implicated in collagenolysis for MMP-1 and MMP-8^36^,^37^,^38^. The “proximity” model is supported by binding studies in which sMT1-MMP had greater affinity for type I collagen than the HPX domain alone (K_D_ = 36 and 527 nM, respectively)^39^.

Distinct regions within the MT1-MMP HPX domain were suggested to interact with the collagen triple-helix, based on changes in NMR signal intensities. Recent interaction studies between the MT1-MMP HPX domain and a series of THPs indicated that key residues were R330, R343, R345, F360, and E392, confirmed by mutational analysis of enzyme activity^17^. The present study has indicated the same overall interaction region within the HPX domain, and specifically R330 and R345 (Figs 3C and S5). Therefore, the current experimental data are in agreement with the binding mode calculated by Zhao *et al*. (pdb 2MQS)^17^.

Highly significant is the shift in THP binding to MT1-MMP compared with MMP-1 (see Fig. 4). This supports a proposed different collagenolytic mechanism for MT1-MMP compared with MMP-1^24^,^40^,^41^. Shifted THP binding is consistent with the recent study mapping THP interactions with the MT1-MMP HPX domain^17^.

Membrane protein function is modulated by the interaction with lipid bilayers. In this respect, different membrane-mimicking environments with specific bilayer properties in terms of thickness, charge, packing, and curvature may influence the structure, orientation, and dynamics of the protein, with a direct effect on its function. The use of DMPC/CHAPS bicelles as a physiologically relevant model of cellular membranes has been previously discussed^29^,^42^. The effect of bicelles on the THP was first evaluated by solution NMR spectroscopy. No interaction between the THP and the membrane-like environment occurred since no variation in signal intensity or chemical shift was visible in the 1D ^1^H NMR spectra. The absence of interaction between the bicelles and the THP was confirmed by CD spectroscopy. CD spectral analysis of the THP indicated that the spectrum and melting point (*T*_m_ value) of the THP showed only minor differences in the presence or absence of bilayer mimics (Figure S15). Thus, different interactions observed with the MT1-MMP HPX domain are not due to interactions of the triple-helix with the bicelles.

Applying docking calculations as performed previously for the MMP-1•THP complex^24^ allowed us to develop a working hypothesis for the structural orientation of MT1-MMP•THP in bilayer membranes (Figure S12). While decrease in signal intensity do not necessarily indicate direct binding interactions, our prior docking studies with MMP-1•THP, which used the same NMR approach^24^, identified interactions within the HPX domain that corresponded extremely well with those observed by X-ray crystallography^35^. Models of the interaction of the MT1-MMP HPX domain alone (Figure S12A and C) and the MT1-MMP HPX•THP complex (Figure S12B and D) with the bilayer are helpful to explain two interesting experimental results. The increase of the signal intensity observed for the residues of HPX interacting with the bilayer upon the addition of THP, and the decrease of the K_M_ for the FRET THP measured when bicelles are present in solution, indicate that the THP and bicelles interplay and modulate the enzymatic activity. The two effects are consistent with the altered binding mode of the enzyme for bicelles and THP when present together in solution. In this respect, the possible steric hindrance between the THP bound to the HPX domain and the bilayer suggested by the analysis of docking calculations is a possible explanation (Figure S12B and D). Although the THP alone has a negligible affinity for the bilayer, the docking models suggest that a disruptive interaction between the *C*-terminal region of the THP and the bilayer may occur allowing a reduction of the affinity constants of the enzyme for both the THP and the bilayer (Figs 3 and S12). It is important to note that our model presents a static picture of a dynamic system, where the HPX domain transiently binds to bicelles with blade III and IV. In fact, the decrease in signal intensity affects a well-defined subset of residues grouped in a distinct surface section of the β-propeller, and is not distributed over all residues of the protein as it would be the case for the formation of a stable complex with high molecular weight. Although the absence of structural data on sMT1-MMP prevents the calculation of a reliable model describing the interaction of the full-length enzyme with the bilayer, the docking calculations of the HPX domain on the selected bilayer model indicated that the binding of blade III and IV of the β-propeller with the membrane is plausible to also occur on the cell surface.

The HPX domain and sMT1-MMP interact in a more specific fashion with bicelles than liposomes. The differences observed for bicelles and liposomes were not due to orientational effects during NMR studies, as the kinetic data were complementary. Based on the NMR data, MT1-MMP binds to bicelles and mammalian cells in a similar fashion (Figs 3 and S8). In turn, liposomes bind to MT1-MMP differently than the bicelles (Figure S7). Most likely the CHAPS in the bicelle mimics cholesterol in the mammalian membrane, where both affect the spacing of the choline-based lipids as well as the fluidity of overall membrane systems.

“Cartoon” mice show craniofacial anomalies, including a shortened head and snout, very large eyes, and a lower average body weight with shortened life span. We found that the cartoon mouse MT1-MMP S466P mutation resulted in increased activity towards triple-helices and decreased activation in the presence of the bilayer membrane. These results are consistent with our recent report on quantification of MT1-MMP cell surface triple-helical peptidase activity^43^. In that study, deletion of the HPX domain, even in the presence of the TM and CT domains, reduced triple-helical peptidase activity. Deletion of the HPX domain also decreased MT1-MMP cell surface collagenolytic activity^44^. It should be noted that the S466P mutation may also have other effects, such as altering MT1-MMP maturation, trafficking, and/or signaling.

Two antibodies raised against the MT1-MMP HPX domain were each found to effectively inhibit cell-based fibrillar collagen degradation (IC_50_ values of 420 nM and 1.56 μM), but were poor inhibitors of sMT1-MMP collagenolysis^39^. These antibodies may act by disrupting the MT1-MMP HPX domain interaction with the cell membrane.

MT1-MMP has been proposed to have numerous cell surface binding partners^2^. CD44 binds to MT1-MMP via blade I of the HPX domain^3^,^45^. Based on our studies, CD44 and collagen cannot bind MT1-MMP at the same time. Using mice carrying null mutations for CD44, Chun *et al*. demonstrated that MT1-MMP collagenolytic activity was not significantly affected by the absence of CD44^46^. This suggests that CD44 is shed, either by MT1-MMP or another protease^2^, prior to MT1-MMP-mediated collagenolysis.

A prior study indicated a requirement of MT1-MMP homodimerization through the HPX domain for efficient hydrolysis of insoluble collagen fiber films^47^. Homodimerization has been proposed to be symmetrical, involving residues D385, K386, T412, and Y436 in blades II and III of the HPX domain^16^, although other studies have suggested that homodimerization requires the outermost strand of blade IV^3^. Homodimerization via blades II and III conflicts with the membrane binding sites demonstrated here. The low level of collagenolytic activity observed with the D385K/T412A/Y436F MT1-MMP mutant^16^ may have been due to disruption of favorable MT1-MMP interaction with the cell surface rather than dimer disruption. Homodimerization may be of weak affinity, and/or only a small fraction of cell surface MT1-MMP may be present as a dimer. Dimerization on the cell surface may regulate MT1-MMP collagenolysis as dimer formation would reduce membrane interaction with MT1-MMP. In solution, MT1-MMP was not found as a dimer^17^ and the HPX domain alone did not form a dimer^26^.

It is known that the MT1-MMP linker is extensively glycosylated^48^, and glycosylation regulates MT1-MMP substrate targeting^49^. Glycosylation may make the linker more constrained due to steric interactions and/or association with the membrane.

Overall, our results agree with the binding mode of the THP previously described^17^,^50^ involving blade I and II of the HPX domain, far away from the HPX domain region interacting with the bilayer. This binding mode is consistent with a plausible sequence of events where the long linker allows MT1-MMP to sandwich the substrate between the CAT and HPX domains during collagenolysis as recently suggested^17^,^50^. Importantly, the sandwiching of the substrate can still occur when the enzyme is associated with bilayers, providing a physiologically relevant collagenolytic mechanism that is clearly distinct from that of MMP-1. These distinct mechanisms of action provide insight into the (a) different collagen preferences of MMP-1 and MT1-MMP and (b) potential design of selective inhibitors based on HPX domain interactions.

## Methods

### NMR spectroscopic analysis of MT1-MMP

Methods for chemically synthesizing ^13^C,^15^N-labeled α1(I)772-786 THP and recombinant production of MMPs in *E. coli* cells have been described^24^. The cDNA encoding the soluble ectodomain of MT1-MMP (Y112-C508; sMT1-MMP), the CAT domain (Y112-K292), and the HPX domain (G315-C508) was amplified by polymerase chain reaction (PCR) and cloned into the pET21 (Novagen) expression vector. The recombinant vector was transformed into *E. coli* strain BL21(DE3), and colonies were selected for ampicillin resistance. The bacteria were grown in LB media. When a cell density corresponding to 0.6 A was reached, the expression of the protein was induced by adding 0.5 mM of isopropyl β-D-thiogalactoside (IPTG) and the incubation at 310 K was continued for another 5 h. sMT1-MMP as well as the isolated domains precipitated in the inclusion bodies, and these were solubilized, after lysis of the cells, in a solution of 8 M urea, 20 mM dithiotreithol (DTT), and 20 mM Tris•HCl (pH 8.0). The solubilized sMT1-MMP and HPX were diluted with a buffer containing 6 M urea, 10 mM CaCl_2_, 0.1 mM ZnCl_2_, 20 mM cysteamine, and 20 mM Tris•HCl (pH 8) and refolded by using a multistep dialysis against solutions containing 50 mM Tris•HCl (pH 8), 4 M urea, 10 mM 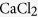, 0.1 mM 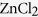, 5 mM β-mercaptoethanol, and 1 mM 2-hydroxyethyl disulfide, against a solution containing 50 mM Tris•HCl (pH 7.2), 2 M urea, 10 mM CaCl_2_, 0.1 mM 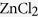, and 0.3 M NaCl, and then against the same solution without urea. The protein was purified by size exclusion chromatography on the HiLoad 26/60 Superdex (GE Biosciences). For the expression of ^15^N-enriched sMT1-MMP and isolated domains, bacteria were grown in minimal medium containing ^15^N-enriched (NH_4_)_2_SO_4_ (Cambridge Isotope Laboratories).

Inactive MT1-MMP was produced by mutation of the active site Glu240 to Ala and removal of the active site 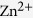 by dialysis^24^. The resonance assignment and solution structure of THP have been previously reported^24^. The isolated CAT and HPX domains and sMT1-MMP have been cloned, expressed, and the resonances assigned by NMR in solution (see Figures S1–3). The assignment of the isolated domains has been obtained analyzing the triple resonance spectra 3D HNCA, 3D CBCA(CO)NH, and 3D HNCACB. The assignment of the resonances of the 2D ^1^H,^15^N-HSQC spectrum of sMT1-MMP has been obtained superimposing the 2D ^1^H, ^15^N-HSQC spectra of the isolated domains with that of sMT1-MMP and confirmed with the triple resonance experiments 3D HNCA and 3D CBCA(CO)NH. NMR experiments were performed at 298 K and acquired on DRX 500 MHz and Bruker AVANCE spectrometers operating at 700, 800, and 950 MHz and equipped with triple resonance cryo-probes. The experiments for the determination of ^15^N longitudinal and transverse relaxation rates and ^1^H-^15^N NOE were recorded at 298 K and 700 MHz on ^15^N-enriched samples of isolated CAT and HPX domains. The ^15^N longitudinal relaxation rates (R_1_) were measured using a sequence modified to remove cross-correlation effects during the relaxation delay^51^. Inversion recovery times ranging between 2.0 and 3000 ms, with a recycle delay of 3.5 s, were used for the experiments. The ^15^N transverse relaxation rates (R_2_) were measured using a Carr-Purcell-Meiboom-Gill (CPMG) sequence^51^,^52^, with delays ranging between 8.5 and 237.4 ms.

Relaxation measurements were also performed on sMT1-MMP. The R_2_ data measured on sMT1-MMP protein were found nosier and less uniform with respect to those of the individual CAT and HPX domains, mainly because of overlap of the resonances. The R_2_ data were measured using a Carr-Purcell-Meiboom-Gill (CPMG) sequence^51^,^52^, with delays ranging between 8.5 and 152.6 ms and a refocusing delay of 450 μsec. An attempt of collecting longitudinal relaxation rates was also performed. However, the relatively fast proteolysis occurring at the long linker affected the reliability of the R_1_ data.

All spectra are processed with the Bruker TOPSPIN software packages and analyzed by the program CARA (Computer Aided Resonance Assignment, CANTINA Verlag, 2004–ETH Zürich)^53^.

The analyses were carried out by adding increasing amount of bicelles (0.5%, 1%, 2%, or 4% bicelles/final volume of the protein solution, w/v) or CHAPS alone (0.1, 0.2, 0.4, 0.8, 1.6, 3.2, 6.4, 9.6, or 12.8 mM) to solutions of MT1-MMP HPX domain. Signal intensities were compared for cross-peaks in 2D ^1^H-^15^N HSQC spectra acquired at 298 K. Bicelles were composed of 1,2-dimyristoyl-sn-glycero-3-phosphocholine (DMPC)/CHAPS (Avanti Polar Lipids, Inc.) in a molar ratio 3:1, and assembled *in situ* by adding DMPC to the protein solution, followed by the addition of CHAPS.

1,2-Dipalmitoyl-*sn*-glycero-3-phosphocholine (DPPC) liposomes were obtained by ultrasound sonication of 25 mg of DPPC in 1 mL of protein buffer.

Human embryonic kidney cells (HEK293T) were grown in DMEM (high glucose, D6546, Sigma) supplemented with L-glutamine, antibiotics (penicillin and streptomycin), and 10% FBS (Gibco) in uncoated 75-cm^2^ plastic flasks and incubated at 310 K, 5% CO_2_ in a humidified atmosphere. Cells were then detached from the culture flask with trypsin-EDTA 0.05% (Gibco) and resuspended in 20 mL DMEM containing 10% FBS to inactivate trypsin. Cells were gently centrifuged (5 min, 800 × g) and washed with PBS (137 mM NaCl, 2.7 mM KCl, 10 mM Na_2_HPO_4_, 1.8 mM KH_2_PO_4_, pH 7.2). Cells were counted with a Burker chamber and 10 μL of the suspension containing 2.0 × 10^6^ cells was added to the MT1-MMP HPX domain solution for the NMR study.

### Kinetic analysis of MT1-MMP

The proenyzme form of sMT1-MMP (designated pro-sMT1-MMP) was obtained from EMD Millipore (catalog # CC1043). The “cartoon” pro-sMT1-MMP included the MT1-MMP sequence from Met1 to Glu532 with a single point mutation, where Ser466 was changed to a Pro. The S466P mutation in MT1-MMP was generated by PCR using the following primers: ctcctagagggccattcatggg. The mutated sequence was then amplified using the following primers: gccgatgctagcgcagaattccggaccatgtctcc and gccgataagctttcactccttcgtccacctcaatg. NdeI and HindIII restriction sites were incorporated to facilitate sub-cloning into a pET28 vector (Novagen). The ligation mixture was used to transform competent cells of *E. coli* DH5α. Plasmids from selected colonies were sequenced and verified for the desired S → P mutation. Once confirmed, protein production was carried out in *E. coli* strain Rosetta(DE3)pLysS. *E. coli* were induced with 1 mM IPTG once an OD600 of 0.5 was reached, and further incubated at 310 K for 2 to 3 h. Recombinant pro-sMT1-MMP S466P was located in the inclusion bodies, which were solubilized in 6 M urea, 150 mM NaCl, 50 mM Tris, pH 8, 5 mM TCEP, 5 mM CaCl_2_, and 1 mM PMSF. Proper refolding was ensured by following a 3 step dialysis in Buffer 1 (1.5 M Urea, 50 mM Tris, pH 8, 150 mM NaCl, 5 mM CaCl_2_, 100 μM ZnCl_2_, 5 mM β-mercaptoethanol, 1 mM 2-hydroxyethyl disulphide, 0.1% Brij 35 (v/v), and 1 mM PMSF), Buffer 2 (50 mM Tris, pH 8, 150 mM NaCl, 5 mM CaCl_2_, 50 μM ZnCl_2_, 5 mM β-mercaptoethanol, 1 mM 2-hydroxyethyl disulphide, 0.1% Brij 35 (v/v), and 1 mM PMSF) and Buffer 3 (50 mM Tris, pH 8, 150 mM NaCl, 5 mM CaCl_2_, 50 μM ZnCl_2_, 0.1% Brij 35 (v/v), and 1 mM PMSF). Each dialysis was performed for 12 h at 277 K and samples were centrifuged, aliquoted, and stored at 203 K till use.

MT1-MMP was activated by incubation of pro-sMT1-MMP in TSB (50 mM Tris, 50 mM NaCl, 10  mM CaCl_2_, 0.05% Brij-35, pH 7.5) with 0.1 μg/mL of rhTrypsin-3 for 1 h at 310 K^54^. After MT1-MMP activation, remaining trypsin-3 activity was quenched by addition of 1 mM AEBSF (R&D Systems) and incubation for 15 min at room temperature. Immediately after activation the enzyme was diluted in cold TSB. Enzyme aliquots were kept on wet ice and used the same day.

A 20% bicelle stock solution was prepared by adding DMPC (Avanti) and CHAPS (Avanti) to TSB to obtain a lipid distribution of 15% and 5% (w/v), respectively. The bicelle mix was then diluted to a final concentration of 20, 5, or 1% with TSB and 2.5 nM of sMT1-MMP or 3 nM of sMT1-MMP S466P was added to the mix.

Liposomes were prepared by adding DPPC (Avanti) to TSB to obtain a stock solution of 25% (w/v). Solubilization was achieved by sonication. The liposome mix was then diluted to a final concentration of 20, 5, or 1% with TSB and 2.5 nM of sMT1-MMP or 3 nM of sMT1-MMP S466P was added to the mix.

Active enzyme concentrations were determined by TIMP-2 titration using Knight substrate^38^. The triple-helical substrate fTHP-9 [(Gly-Pro-Hyp)_5_-Gly-Pro-Lys(Mca)-Gly-Pro-Gln-Gly~Cys(Mob)-Arg-Gly-Gln-Lys(Dnp)-Gly-Val-Arg-(Gly-Pro-Hyp)_5_-NH_2_, where Hyp = 4-hydroxyproline, Mca = (7-methoxycoumarin-4-yl) acetyl, Mob = 4-methoxybenzyl, and Dnp = 2,4-dinitrophenyl] was synthesized as described previously^38^,^55^. Substrate stock solutions were prepared in TSB containing 0.5% DMSO. MT1-MMP assays were conducted in TSB by incubating a range of substrate concentrations (20 to 0.04 μM) with 7 nM enzyme at 310 K. Fluorescence was measured on a multiwell plate fluorimeter (Biotek Synergy H1) using λ_excitation_ = 324 nm and λ_emission_ = 405 nm. Rates of hydrolysis were obtained from plots of fluorescence *versus* time, using data points from the linear portion of the hydrolysis curve alone. The slope from these plots was divided by the fluorescence change corresponding to complete hydrolysis and then multiplied by the substrate concentration to obtain rates of hydrolysis in units of μM/sec. The relationship between the rate of hydrolysis and substrate concentration for the MT1-MMP/fTHP-9 pair for which individual kinetic parameters were determined was found to follow the Michaelis-Menten model. Kinetic parameters were evaluated by Lineweaver-Burk, Eadie-Hofstee, and Hanes-Woolf analyses. Data were additionally analyzed using nonlinear regression, one-site hyperbolic binding model with GraphPad Prism 5 software. All the values reported are mean ± SD (n = 3).

### Modeling of the complexes with membrane bilayers

The models of the complex between either the isolated HPX domain or the HPX domain bound to the THP and the membrane-mimicking environment were obtained performing docking calculations with the program HADDOCK 2.2 56. The X-ray crystallographic structure of the HPX domain of MT1-MMP (pdb 3C7X), and the NMR-derived complex between the HPX domain and THP (pdb 2MQS), were implemented as input coordinates. The coordinates of DMPC bilayer, with 360 DMPC molecules, were generated using the Web-Based Graphical User Interface for CHARMM (http://www.charmm-gui.org)^57^ and then equilibrated with GROMACS^58^. The topology and parameters files for the DMPC molecule were generated by PRODRG (http://davapc1.bioch.dundee.ac.uk/prodrg/)^59^. The limit on lipid molecules allowed in HADDOCK simulations was raised in order to implement bilayers in the docking calculations (i.e., MAXTREE was set to 400). In the docking calculations the residues of the HPX domain were ambiguously restrained to ten alterative molecules constituting the membrane bilayer. Semi-flexibility was allowed to both the interacting portions of the protein and the selected molecules of the bilayer. The constant of the intermolecular interactions for rigid body docking was scaled down to 0.01, and the number of molecular dynamics steps for the first two parts of the protocol (rigid-body high temperature dynamic and slow cooling annealing in the semi-flexible refinement) was set to 0 to favor the interaction with the lipids. During the rigid docking calculations, 1000 complexes were generated, then 200 structures were selected for the semi-flexible simulated annealing in torsion angle space, and finally refined in Cartesian space with explicit solvent.

A model of sMT1-MMP was obtained using Modeller^60^. The X-ray structures of the isolated domains (pdb 3MA2 and 3C7X for CAT and HPX, respectively) and the reciprocal orientation of the two domains in the X-ray structure of MMP-12 (pdb 3BA0) were used as a template.

## Additional Information

**How to cite this article**: Cerofolini, L. *et al*. Bilayer Membrane Modulation of Membrane Type 1 Matrix Metalloproteinase (MT1-MMP) Structure and Proteolytic Activity. *Sci. Rep.* **6**, 29511; doi: 10.1038/srep29511 (2016).

## Supplementary Material

Supplementary Information

## Figures and Tables

**Figure 1:**
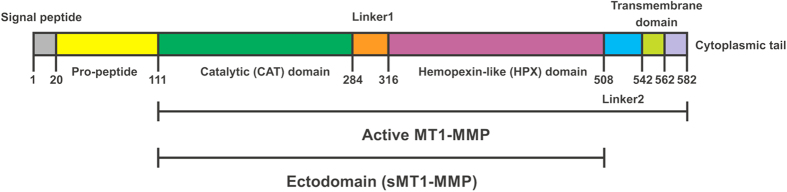
Multidomain structure of MT1-MMP. Residues numbers for each domain are indicated.

**Table 1:** Kinetic parameters for fTHP-9 hydrolysis by MT1-MMP.

MT1-MMP	Lipid	K_M_(μM)	k_cat_/K_M_ (sec^−1^M^−1^)	k_cat_(s^−1^)
sMT1-MMP	None	4.23 ± 0.52	69,720 ± 3,317	0.30 ± 0.05
“	1% Bicelle	7.08 ± 0.41	106,700 ± 2,504	0.75 ± 0.05
“	5% Bicelle	8.85 ± 0.96	76,250 ± 2,716	0.67 ± 0.05
“	20% Bicelle	11.05 ± 1.02	60,710 ± 5,838	0.80 ± 0.26
“	1% Liposomes	8.2 ± 0.3	92,910 ± 6,768	0.76 ± 0.07
“	5% Liposomes	15.6 ± 1.52	67,300 ± 4,771	1.05 ± 0.18
“	20% Liposomes	11.2 ± 0.3	57,580 ± 3,770	0.80 ± 0.20
sMT1-MMP S466P	None	4.27 ± 0.76	79,750 ± 6,118	0.33 ± 0.037
“	1% Bicelle	4.98 ± 0.97	95,150 ± 2,328	0.47 ± 0.081
“	5% Bicelle	7.3 ± 0.45	62,570 ± 1,580	0.45 ± 0.017
“	20% Bicelle	11.18 ± 2.24	52,260 ± 13,900	0.63 ± 0.03
“	1% Liposomes	3.43 ± 0.12	104,200 ± 1,337	0.35 ± 0.009
“	5% Liposomes	7.15 ± 0.48	80,900 ± 9,324	0.58 ± 0.09
“	20% Liposomes	6.18 ± 0.23	77,920 ± 2,551	0.48 ± 0.03
