# Supplementary material for: Bilayer Membrane Modulation of Membrane Type 1 Matrix Metalloproteinase (MT1-MMP) Structure and Proteolytic Activity
Source: Sci Rep. 2016 Jul 13;6:29511. doi: 10.1038/srep29511 (PMC4942797; doi:10.1038/srep29511)
Supplement: Supplementary Information [file srep29511-s1.doc]

**Bilayer Membrane Modulation of Membrane Type 1 Matrix Metalloproteinase (MT1-MMP) Structure and Proteolytic Activity**

Linda Cerofolini,^1^ Sabrina Amar,^2^ Janelle L. Lauer,^3^ Tommaso Martelli,^4,5^ Marco Fragai,^4,5^ Claudio Luchinat,^4,5,*^ and Gregg B. Fields^2,6,7,*^

^1^Giotto Biotech S.R.L., Via Madonna del Piano 6, 50019 Sesto Fiorentino (FI), Italy.

^2^Department of Chemistry & Biochemistry, Florida Atlantic University, 5353 Parkside Drive, Jupiter, FL 33458, USA

^3^Max Planck Institute of Molecular Cell Biology and Genetics, Pfotenhauerstrasse 108,

01307, Dresden, Germany

^4^CERM, University of Florence, Via Luigi Sacconi 6, 50019, Sesto Fiorentino (FI), Italy

^5^Department of Chemistry “U. Schiff”, University of Florence, via della Lastruccia 3, 50019, Sesto Fiorentino (FI), Italy

^6^Department of Chemistry, The Scripps Research Institute/Scripps Florida, 130 Scripps Way, Jupiter, FL 33458, USA

^7^Departments of Chemistry and Biology, Torrey Pines Institute for Molecular Studies, 33458, Port St. Lucie, FL 34987, USA

*To whom correspondence should be addressed:

Claudio Luchinat, CERM, University of Florence, Via Luigi Sacconi 6, 50019, Sesto Fiorentino (FI) Italy, E-mail: claudioluchinat@cerm.unifi.it

Gregg B. Fields, Florida Atlantic University, 5353 Parkside Drive, Jupiter, FL 33458 USA, E-mail: fieldsg@fau.edu.

**SUPPLEMENTARY MATERIAL**


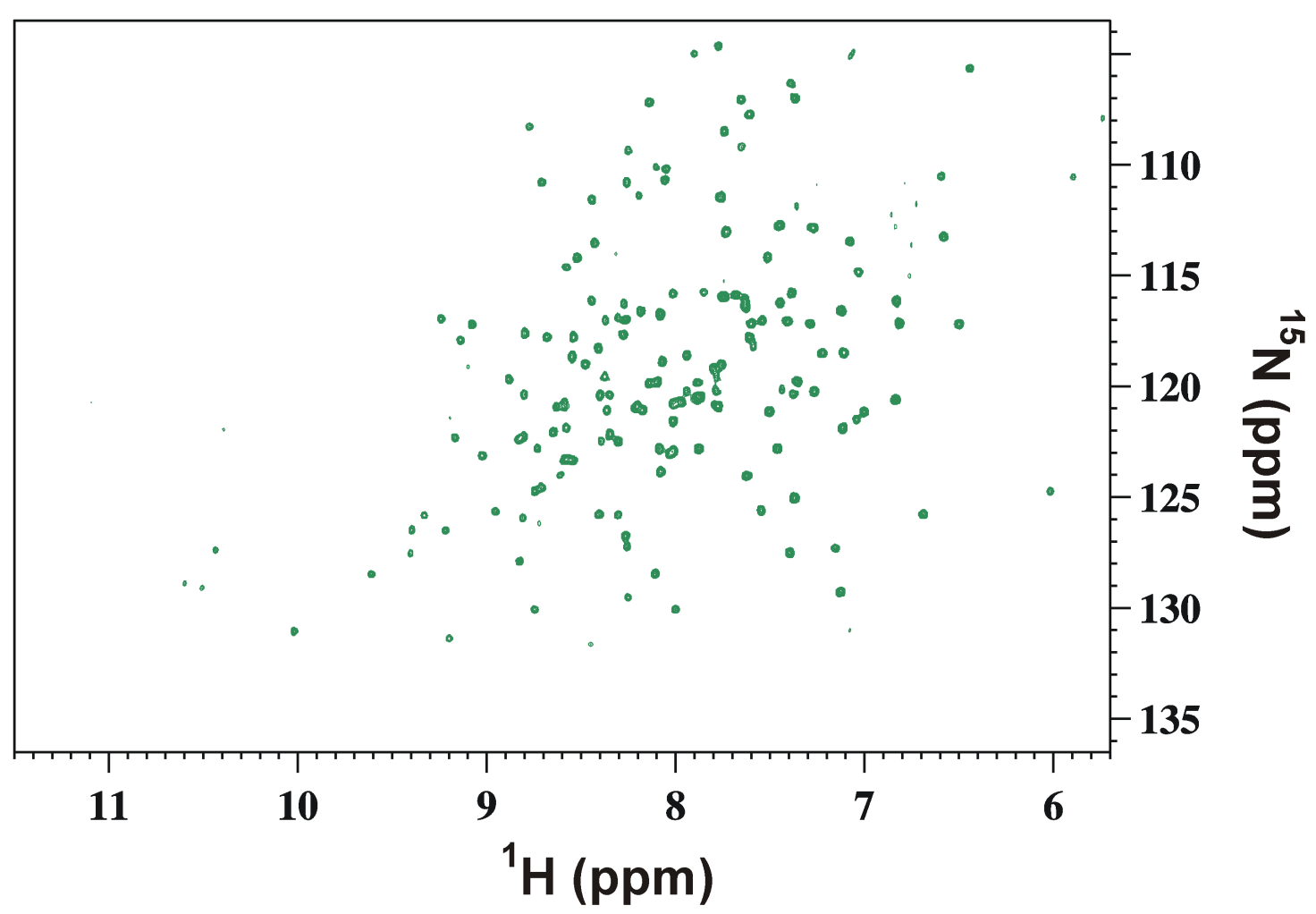


**Figure S1**: 2D ^1^H-^15^N-TROSY-HSQC spectra of ^1^H,^15^N-labeled CAT domain of MT1-MMP.


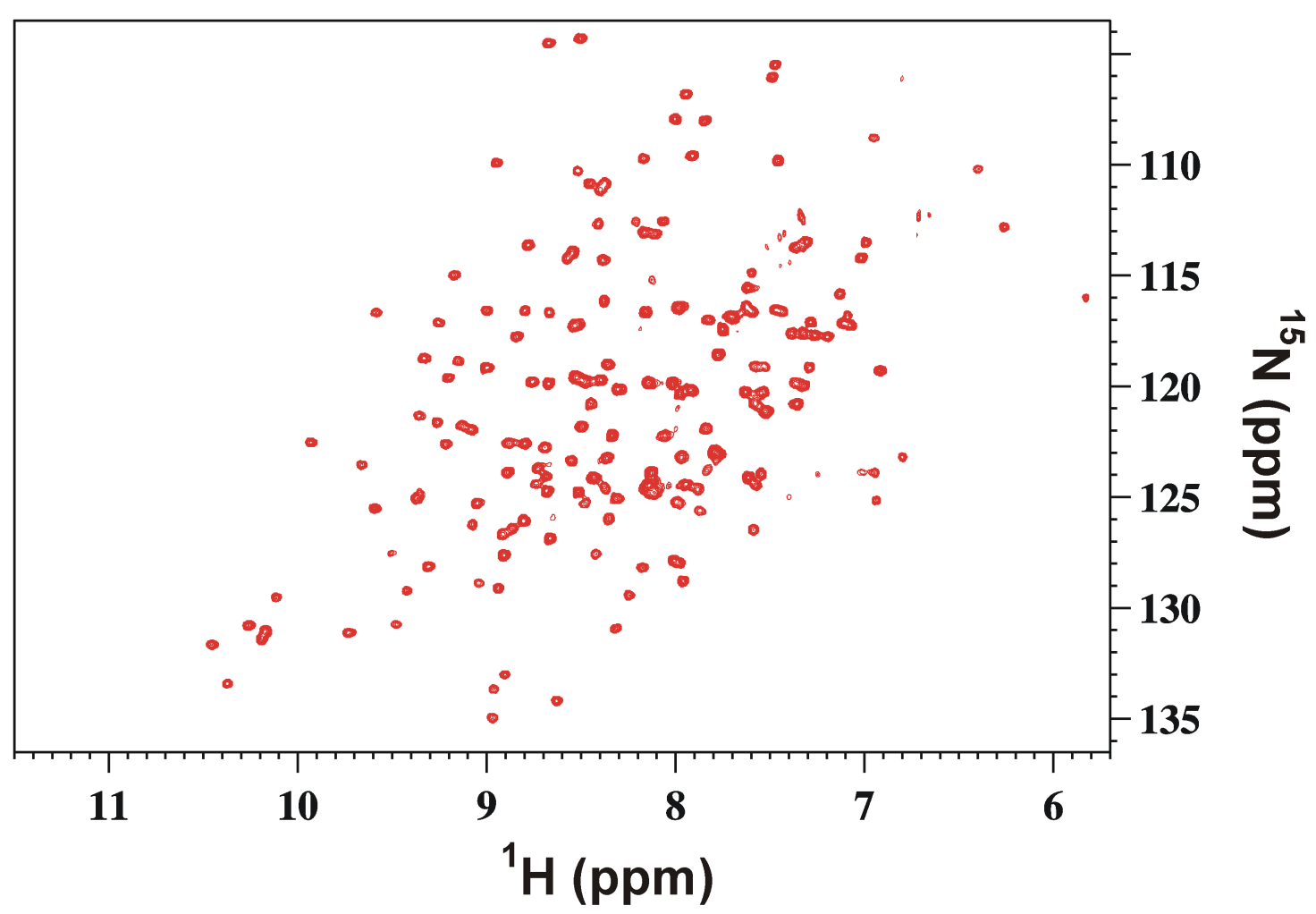


**Figure S2**: 2D ^1^H-^15^N-TROSY-HSQC spectra of ^1^H,^15^N-labeled HPX domain of MT1-MMP.


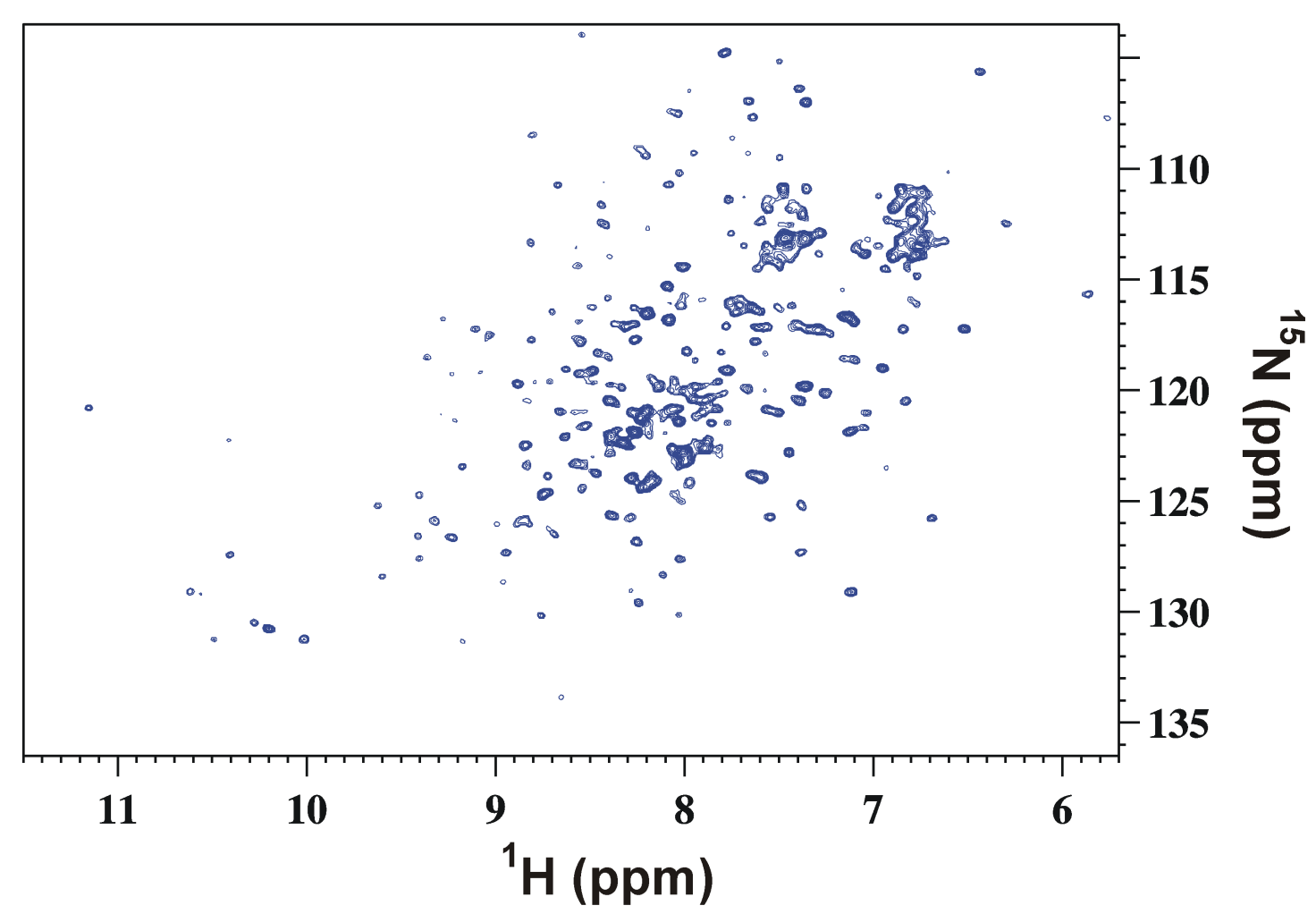


**Figure S3**: 2D ^1^H-^15^N-TROSY-HSQC spectra of ^1^H,^15^N-labeled sMT1-MMP.


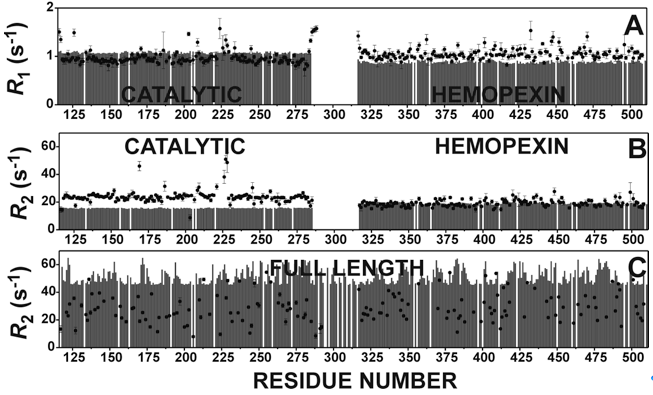


**Figure S4**: Comparison of NMR relaxation data for sMT1-MMP. The calculated (grey bars) and experimental (filled circles) backbone ^15^NH R_1_ and R_2_ values for the isolated CAT and HPX domains of MT1-MMP are shown in panels A and B, respectively. The ^15^NH R_2_ values for sMT1-MMP are shown in panel C. The agreement between experimental and calculated R_1_ and R_2_ values for the isolated domains is good if a slight tendency of the CAT domain to aggregate is taken into account. Conversely, for sMT1-MMP, the experimental R_2_ values (panel C) are sizably lower than the ones calculated for the rigid modeled structure of MT1-MMP.


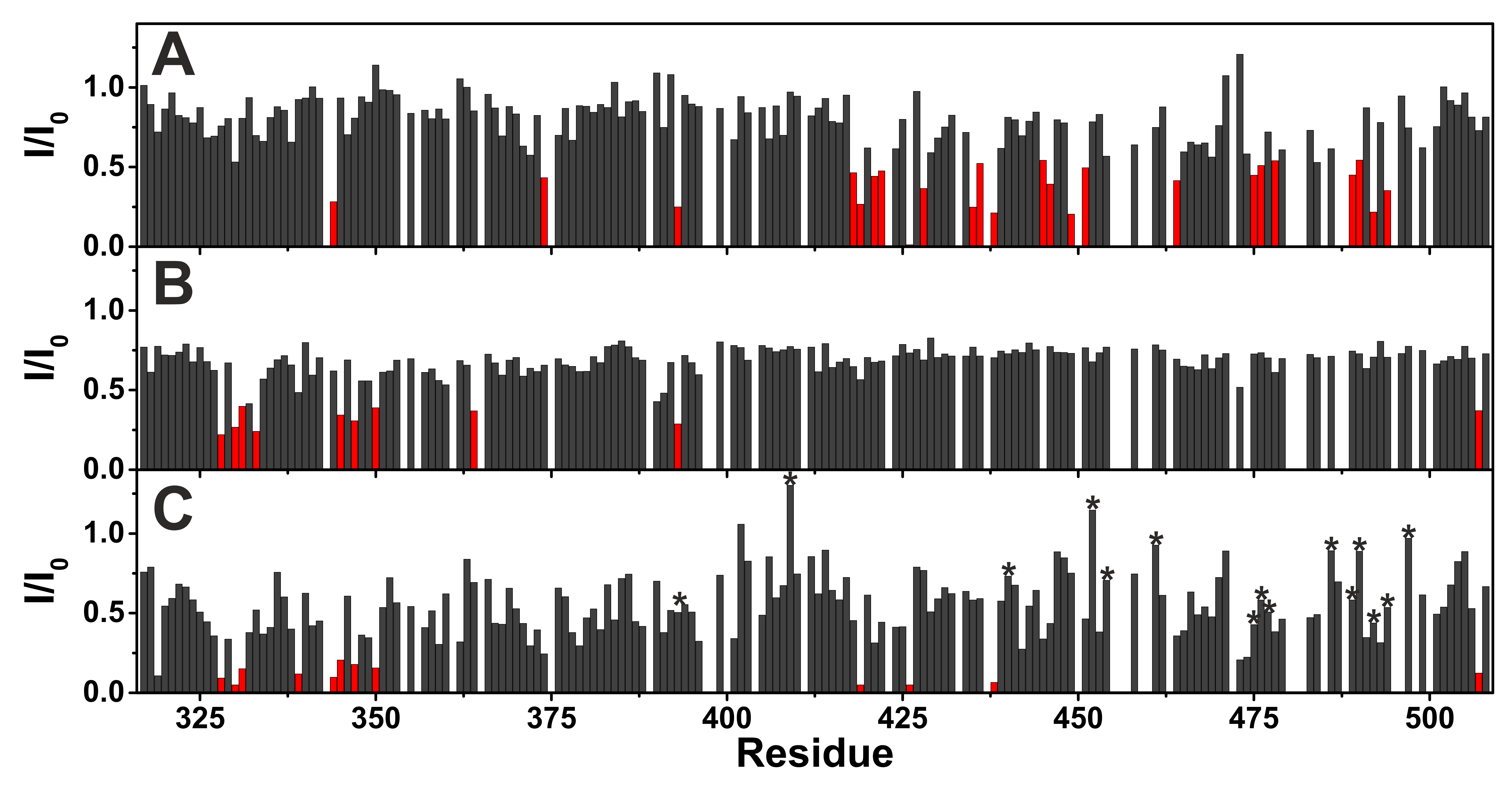


**Figure S5.** Intensity changes per residue of the HPX domain in the presence of 1% w/v bicelles (panel A), THP (molar ratio HPX:THP = 1:0.5) (panel B), and 1% w/v bicelles and THP (molar ratio HPX:THP = 1:1) (panel C). The residues exhibiting the largest decrease in signal intensity in the presence of bicelles (V344, R374, A393, A418, L419, W421, M422, K426, Y428, Y435, Y436, F438, V445, D446, Y449, K451, R464, T475, Y476, Y478, Q489, K490, K492, and E494), THP (M328, R330, G331, M333, R345, N347, M350, L364, A393, and G507) and bicelles and THP (M328, G331, R330, R339, V344, R345, N347, M350, L419, F438 and G507) have been highlighted in red. The intensity of the signals belonging to the HPX domain before the addition of bicelles and THP was taken as reference (intensity = 1). The residues exhibiting an increase in signal intensity after the addition of THP in the presence of bicelles have been marked with a star.

**
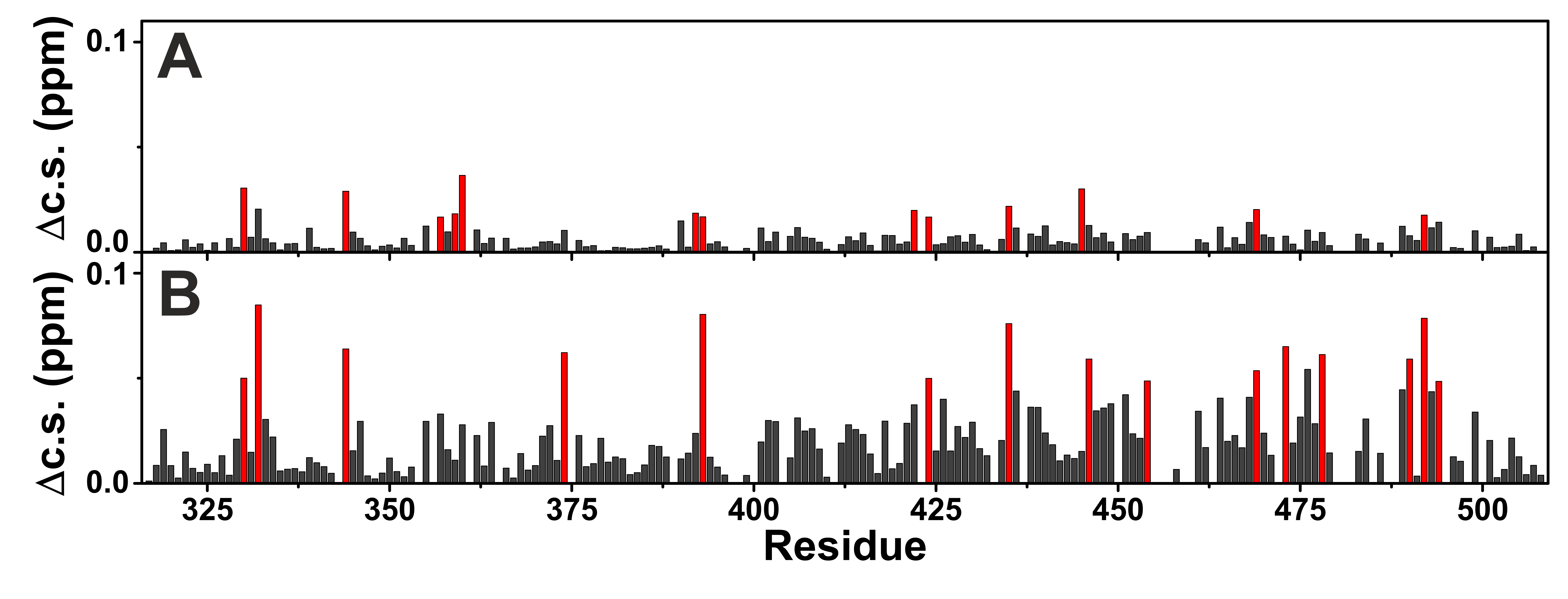
**

**Figure S6.** Chemical shift variations per residue of the HPX domain in the presence of 1% w/v bicelles (panel A), and 1% bicelles and THP (molar ratio HPX:THP = 1:1) (panel B). The residues exhibiting the largest variations in chemical shift in the presence of bicelles (R330, V344, I357, Q359, F360, A393, M422, N424, Y435, V445, G469, and K492) and bicelles and THP (R330, E332, V344, R374, A393, N424, Y435, D446, K454, G469, V473, Y478, K490, K492 and E494) have been highlighted in red.

**
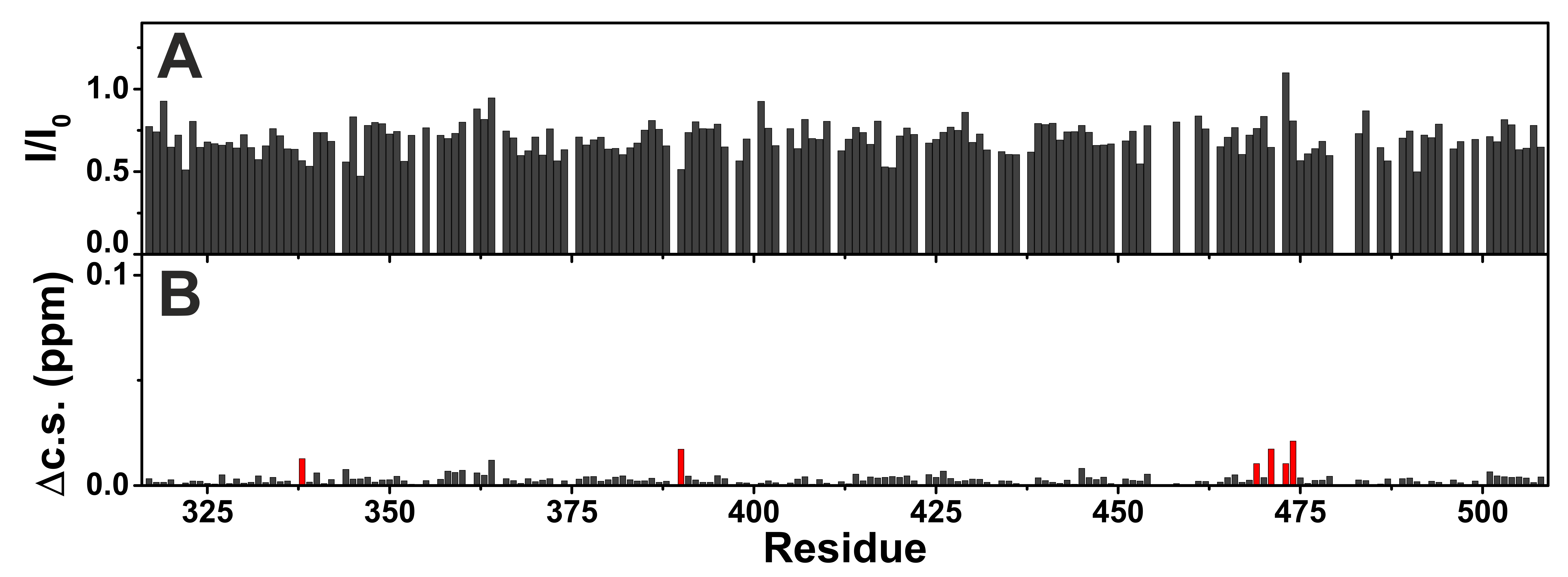
**

**Figure S7**. Intensity changes (panel A) and chemical shift variation (panel B) per residue of the MT1-MMP HPX domain in the presence of 1% w/v DPPC liposomes. The residue exhibiting the highest chemical shift variation (E338, F390, G469, D471, V473, and F474) have been highlighted in red.


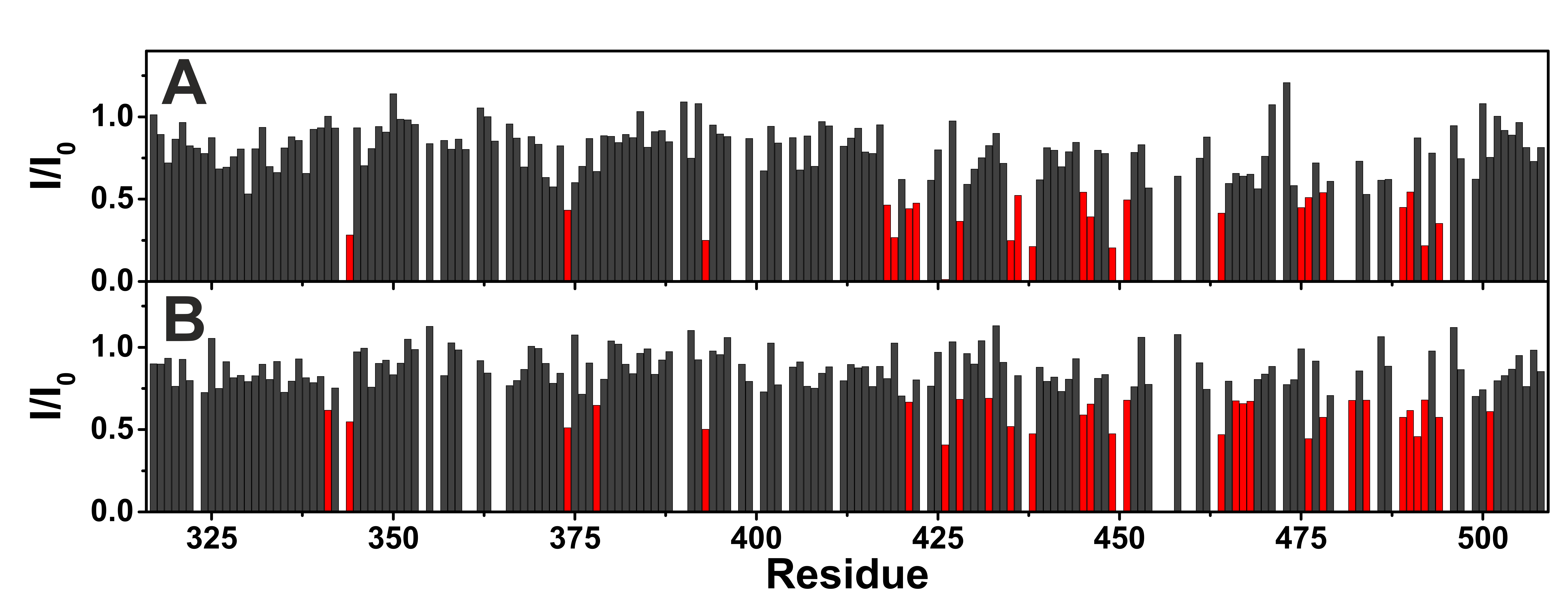


**Figure S8.** Intensity changes per residue of the MT1-MMP HPX domain in the presence of 1% w/v bicelles (panel A) or 10 μL of a pellet containing 2.0 x 10^6^ HEK293T cells (panel B). The residues exhibiting the largest decrease in signal intensity in the presence of bicelles (V344, R374, A393, A418, L419, W421, M422, K426, Y428, Y435, Y436, F438, V445, D446, Y449, K451, R464, T475, Y476, Y478, Q489, K490, K492, and E494) and in the presence of cells (F341, V344, R374, K378, A393, W421, K426, Y428, G432, Y435, F438, V445, D446, Y449, K451, R464, S466, F467, M468, Y476, Y478, K482, W484, Q489, K490, L491, K492, E494 and A501) have been highlighted in red. The intensity of the signals belonging to the HPX domain before the addition of bicelles or cells was taken as reference (intensity = 1).


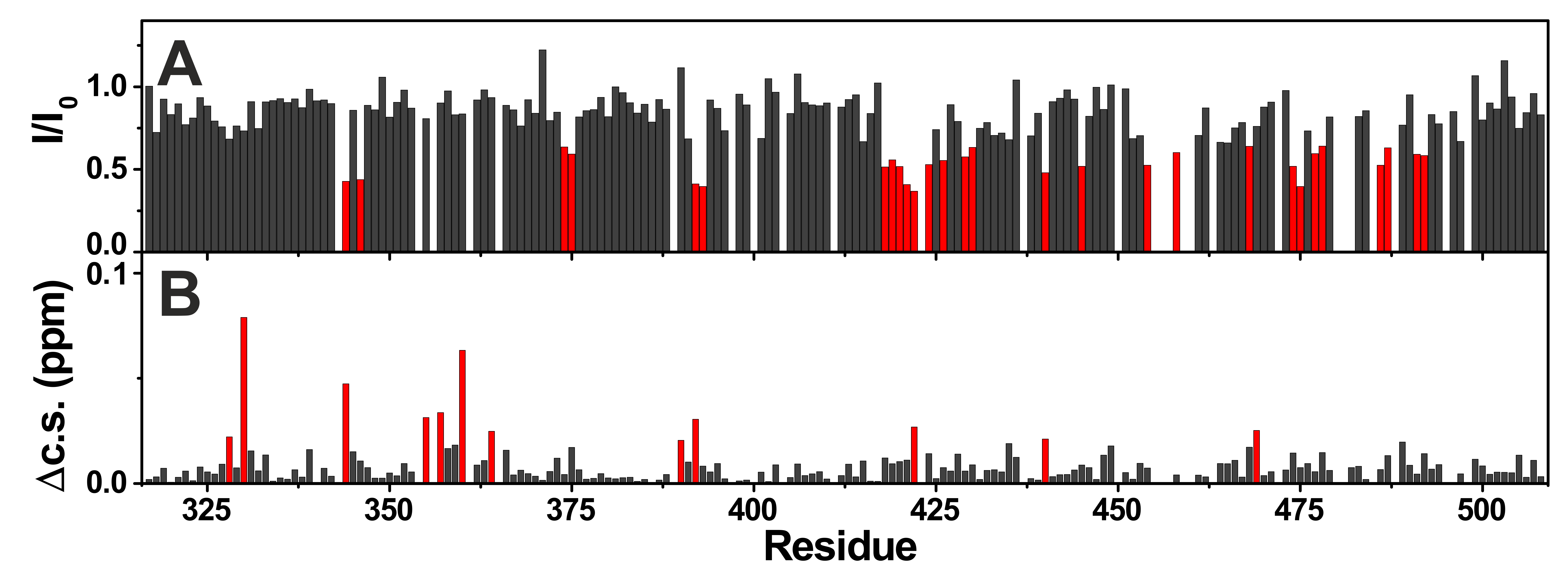


**Figure S9.** Intensity changes (panel A) and chemical shift variation (panel B) per residue of the MT1-MMP HPX domain in the presence of 3.2 mM CHAPS. The residues exhibiting the largest decrease in signal intensity (V344, N346, R374, K375, E392, A393, A418, L419, F420, W421, M422, N424, K426, F429, F430, E440, V445, K454, G458, M468, F474, T475, F477, Y478, F486, N487, L491, and K492) and chemical shift variation (M328, R330, V344, M355, I357, Q359, F360, L364, F390, E392, M422, E440, and G469) have been highlighted in red.


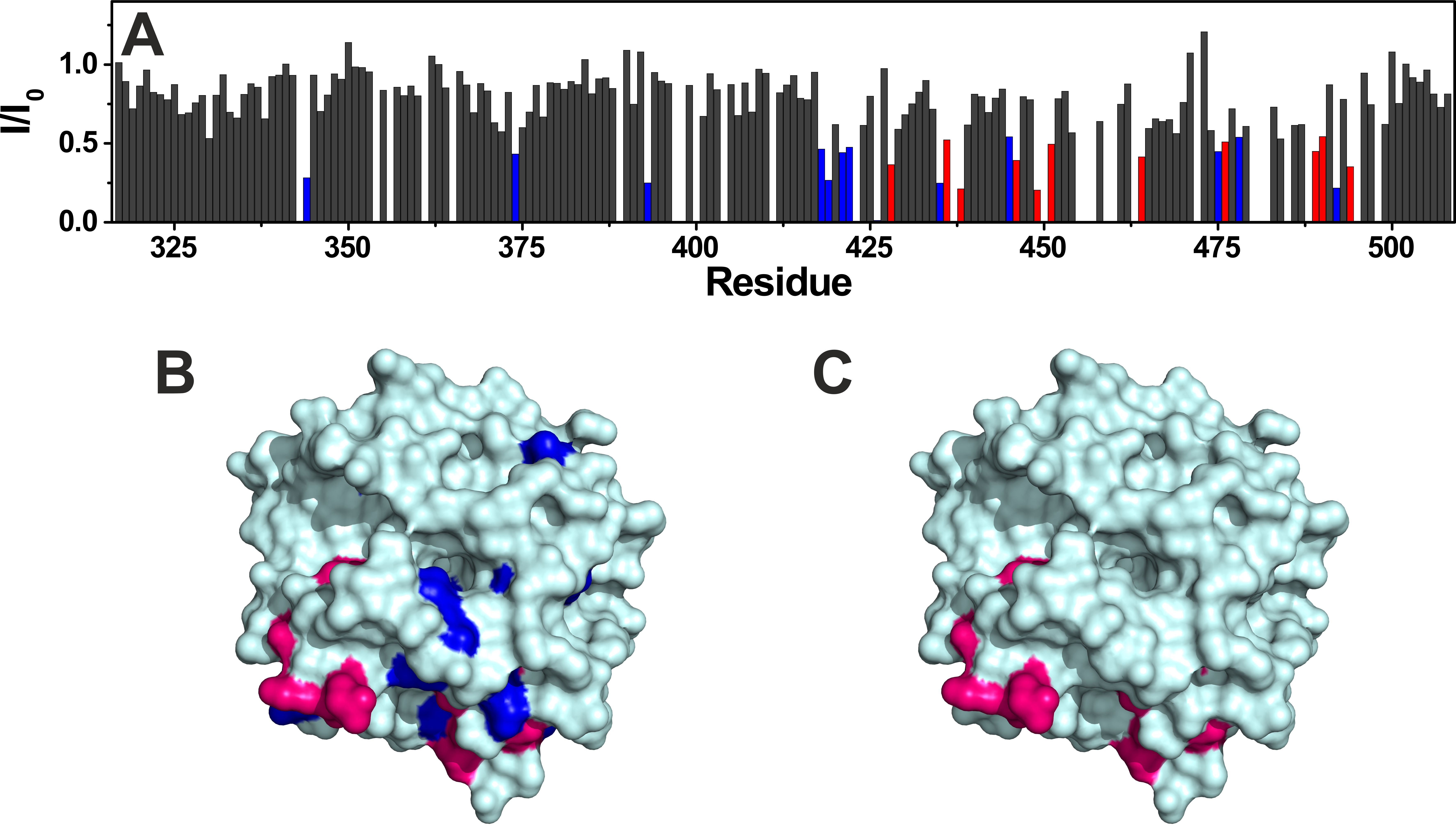


**Figure S10.** Intensity changes per residue of the MT1-MMP HPX domain in the presence of 1% w/v bicelles. The residues experiencing decreases in signal intensity also in the presence of CHAPS alone (3.2 mM) are colored in blue (V344, R374, A393, A418, L419, W421, M422, K426, Y435, V445, T475, Y478, and K492). The residues selectively affected by bicelles are colored in red (Y428, Y436, F438, D446, Y449, K451, R464, Y476, Q489, K490, and E494).


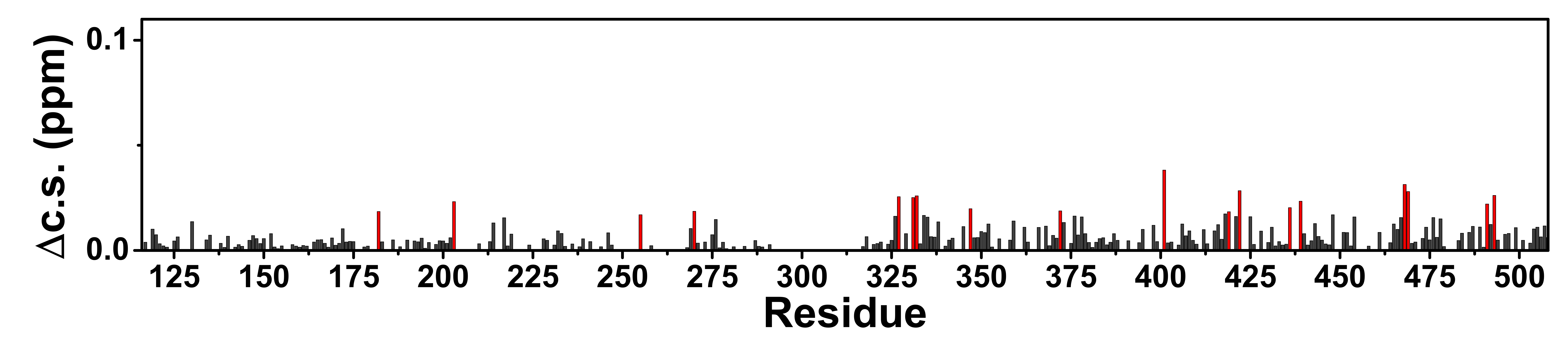


**Figure S11.** Chemical shift variations per residue of sMT1-MMP in the presence of 2% w/v bicelles and THP (molar ratio sMT1-MMP:THP = 1:2, black bars). The residues exhibiting the highest effect after the addition of THP (A182, Y203, A255, V270, A327, G331, E332, N347, Y372, K401, L419, M422, Y436, N439, M468, G469, L491, V493) have been highlighted in red.

**Figure S12.** Docking models of the HPX domain interacting with the membrane bilayer (panel A and C) and complexes of HPX•THP•membrane (panel B and D). All the docking calculations were performed with the program HADDOCK 2.2. The residues experiencing the largest decrease in signal intensity in the presence of 1% bicelles have been highlighted in magenta. In panel A and B, the structural models were obtained when the residues experiencing a decrease in signal intensity in the presence of CHAPS alone were included in the calculations. In panel C and D, the structural models were obtained when the residues experiencing a decrease in signal intensity in the presence of CHAPS alone were not included in the calculations.

**
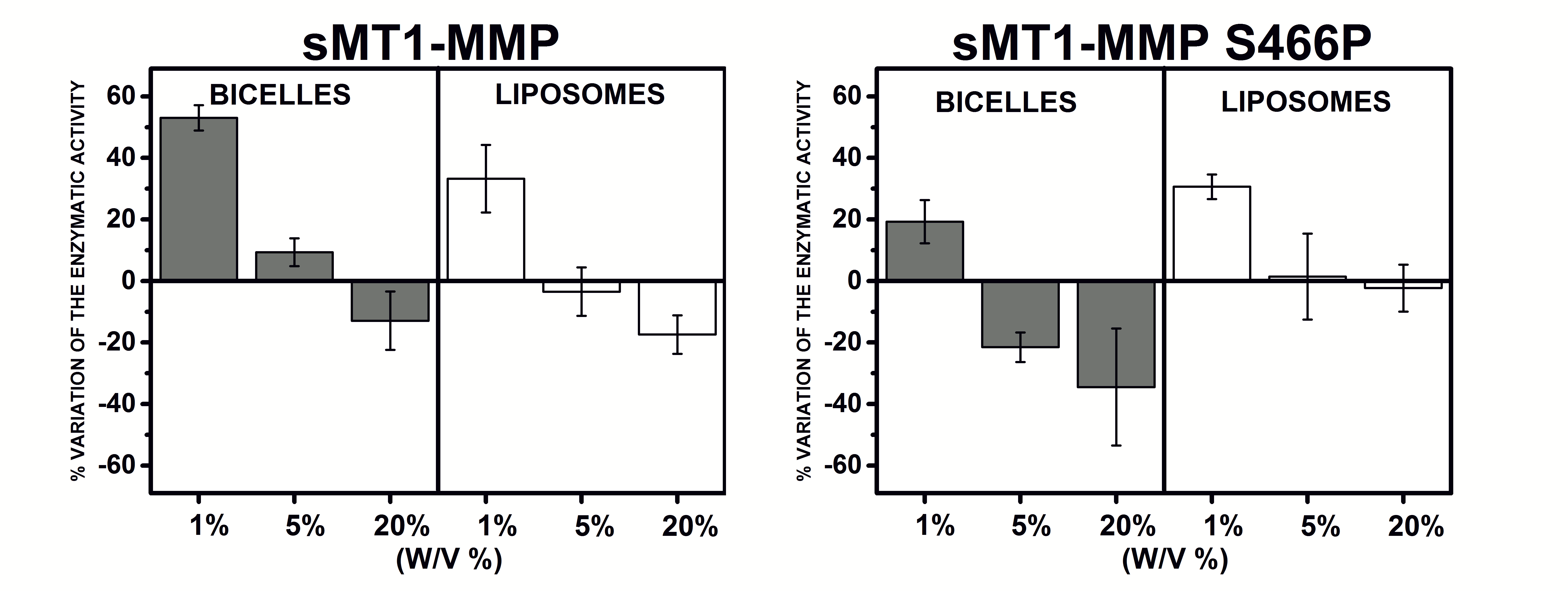
**

**Figure S13.** Graphical representation of the variation of the enzymatic activity of wild type and S466P sMT1-MMP in the presence of different concentrations of bilayer mimics.

**Figure S14**: CD spectra of sMT1-MMP (blue) and sMT1-MMP S466P (red), 50 scans, at 0.2 μg/μL in TSB. Upper panel shows full spectrum, lower panel focuses in on λ = 200-250 nm and CD signal from 1 to -2 mdeg.

**Figure S15.** (A) CD spectra of THP in buffer (pink) and 5% bicelle (red). (B) Melting curves for THP in buffer (pink) and 5% bicelle (red). For each condition, the melting temperature (*T*_m_) was 53.8 and 52 °C, respectively. (C) CD spectra of THP in buffer (pink) and 5% liposomes (red). For each condition, the *T*_m_ was 53.8 and 53 °C, respectively.
